# Original article: adolescent dietary patterns derived using principal component analysis and neuropsychological functions: a cross-sectional analysis of Walnuts Smart Snack cohort

**DOI:** 10.1007/s00787-024-02577-6

**Published:** 2024-09-18

**Authors:** Nicolas Ayala-Aldana, Ariadna Pinar-Martí, Marina Ruiz-Rivera, Sílvia Fernández-Barrés, Dora Romaguera, Jordi Casanova-Mollà, Nuria Solà-Valls, Jordi Julvez

**Affiliations:** 1 Clinical and Epidemiological Neuroscience (NeuroÈ̇pia), Institut d′Investigació̇ Sanità̇ria Pere Virgili (IISPV), Reus, Spain; 2 ISGlobal, Barcelona, Spain; 3 Institut d’Investigació Sanitaria Illes Balears (IdISBa), Palma, Spain; 4 Salut Sant Joan Reus - Baix Camp, Reus, Spain; 5 University of Barcelona, Catalonia Barcelona, Spain; 6 Universitat Pompeu Fabra (UPF), Catalonia Barcelona, Spain; 7 CIBER Fisiopatologí̇a de la Obesidad y Nutrició̇n (CIBEROBN), Instituto de Salud Carlos III, Madrid, Spain; 8 Universitat Rovira i Virgili, Facultat de Medicina i Ciències de la Salut, Human Nutrition Unit, Reus, Spain

**Keywords:** Dietary patterns, PCA, Neuropsychological function, Attention, Emotional recognition, Adolescence

## Abstract

A balanced diet is relevant for neuropsychological functioning. We aimed to analyze the association between dietary patterns and neuropsychological outcomes in a sample of healthy adolescents of the Walnuts Smart Snack (WSS) cohort from Barcelona city. We performed principal components analysis (PCA) to determine dietary patterns in the adolescent sample using a food frequency questionnaire (60 items). Multiple linear regression models were performed to analyze the association between PCA dietary patterns with neuropsychological outcomes: Strengths and Difficulties (SDQ) externalizing and internalizing scores, Attention Network Test (ANT) Impulsivity Index and Emotional Recognition Task (ERT) scores. We additionally adjusted the models for child sex, age, body mass index (BMI), physical activity and maternal education. Six dietary patterns were identified in PCA analyses. “low consumption of calorie-dense foods” dietary pattern had a negative association (protective) with the both SDQ outcomes (p value < 0.001) and “Nuts” dietary pattern showed a negative (protective) association with impulsivity index $\:{(\beta\:}_{1}$= −24.60, 95% CI = −36.80, −12.41, p value < 0.001). Overall, our main results suggest that healthy dietary patterns, including higher intakes of “nuts” and a preference of “low consumption of calorie-dense foods” dietary patterns, could provide a beneficial association with neuropsychological functions during the adolescence period. The associations may include improvements of externalizing and internalizing problem symptoms, and impulsivity.

**Supplementary Information:**

The online version contains supplementary material available at 10.1007/s00787-024-02577-6.

## Introduction

The well-being in the human life cycle depends on maintaining brain health. An inadequate diet is a substantial risk factor for mental health disorders in adults and in the young populations [1, 2]. There are many nutrients and micronutrients in the diet that are essential for brain development, protection and functioning in early stages of life such as lipids, proteins, minerals, and vitamins [3].

During childhood and adolescence, the brain exhibits sensitive phases when experience, environment and nutrition may greatly modify its functional and structural characteristics [4]. Specifically, neuropsychological functions such as attention, impulsivity regulation and emotional recognition are highly developing during adolescence [5–7]. During this period, symptoms related to attention-deficit hyperactivity disorder (ADHD) may hinder the recognition of internal emotions, task execution, academic performance, and social interactions [8–10].

It is crucial to find ways to support neuropsychological development and academic achievement in childhood and adolescence, given that poor neuropsychological performance and low academic achievement in childhood are associated with an increased risk of adult obesity, unemployment, and vulnerable socioeconomic condition [11, 12]. Neuropsychological development and function are influenced by nutrition. Nutrients are relevant components of enzyme systems in the cerebral cortex and provide the essential building blocks for cell division, DNA synthesis, neurotransmitter and hormone metabolism, and cell growth [13, 14]. Inadequate levels of whole foods (i.e. unprocessed), such as those rich in micronutrients (iron, zinc, B12) and long-chain polyunsaturated fatty acids (PUFAs), may result in long-term functional issues like memory loss, ADHD symptoms, depression, or anxiety problems [15]. On the other hand, excessive consumption of junk food in the young population has been related to the development of alcohol consumption, drug abuse, impulsivity and ADHD symptoms [16]. Therefore, an adequate and balanced diet is essential for proper brain development and neuropsychological function [17, 18].

The cornerstone of efforts for the primary prevention of many of the risk factors for neuropsychological impairment is lifestyle and dietary changes [19]. Due to the correlation of dietary preferences, isolating the ‘pure effect’ of each individual food is difficult, making it challenging to investigate the relationship between the dietary intake of each food/nutrient and neuropsychological functions. Sophisticated approaches for determining food patterns include principal components analysis (PCA), a type of dimensionality reduction technique [20]. To find hidden patterns in the data, it uses correlations between food intake or preferences. Aggregation-based PCA provides a unique or multiple factor solution and reduces dietary variables [21]. As an example of research using this innovative technique, a previous study on a population aged 10 to 15 years used PCA to identify dietary patterns, resulting in ‘High protein,’ ‘High fat,’ and ‘High salt-oil’ patterns. The ‘High protein’ pattern score was significantly associated with higher mathematics test scores. Conversely, the ‘High fat’ pattern score was significantly associated with lower mathematics and vocabulary test scores [22]. Another cross-sectional study demonstrated that the high-energy dietary pattern (noodles, eggs, processed meat and fish snacks) was negatively associated with general cognitive ability, perceptual reasoning, and processing speed in adolescent population [23].

Data-driven food patterns of healthy adolescents and their relationships with neuropsychological functions are poorly understood. Indeed, there is limited research of these factors among teenagers, which is an important period of brain development and function. Thus, in this cross-sectional study, we aimed to examine the association between dietary preferences and neuropsychological functions such as externalizing and internalizing regulations, impulsivity and emotion recognition among a healthy adolescent population using the Walnuts Smart Snack (WSS) cohort.

We hypothesized that a balanced diet with a healthy pattern of nutrition would relate to better neuropsychological performance in the adolescent population.

## Materials and methods

### Study design and participants

The current study is based on a cross-sectional design using baseline information from the Walnuts Smart Snack (WSS) trial [24]. The purpose of this intervention was to determine if dietary supplementation with 30 g of raw walnut kernels per day for six months resulted in beneficial improvements in cognitive and socioemotional development when compared to a control group of healthy adolescents [25]. The study targeted adolescents aged 11–16 years attending regular schools in Barcelona. Although the initial protocol aimed to recruit 12–15-year-olds, the inclusion criteria were expanded to accommodate slightly younger and older students willing to participate, as facilitated by the schools. Exclusion criteria comprise individuals who regularly consume omega-3 PUFA supplements, eat walnuts daily, or have allergies to walnuts or gluten. Additionally, participants were excluded if they reported lactose intolerance or allergies to cereals, dried fruits, peanuts, soy, sesame, or sulfites, due to potential traces of these substances in walnut packages as a result of walnut industry practices. Participants with neuropsychological disorders were not excluded. In our study, there are only 3 cases of dyslexia and 1 case of attention deficit without hyperactivity. The rest of the cohort population in this analysis does not report neuropsychological or mental health disorders.

Over a year (2015–2016), we recruited 771 participants from 11 high schools in Barcelona which were evenly distributed geographically. We invited both public and private schools to participate in the project, and our goal was to include at least one high school per municipal district. Participants completed several neuropsychological tests and provided information on their lifestyle and dietary preferences prior to randomization. All information about the clinical trial procedure is described in the WSS protocol [24]. Participants who had complete baseline data on the food frequency questionnaire and the neuropsychological tests were eligible for the present study (*n* = 643). The trial study received permission from Parc Salut Mar’s Clinical Research Ethics Committee (approval number: 2015/6026/I).

A fieldwork technician provided questionnaires to the adolescent participants and other questionnaires were given to the parents to complete at home and return to us through the school instructors. Additionally, the technician asked about dietary habits using a food-frequency questionnaire (FFQ) of 60 food items adapted to the adolescent age from a validated questionnaire for the Spanish population [26, 27]. The adaptation consisted on increasing the portion sizes for some items (from small to medium size for meat, fish, vegetables, rice, pasta, and ultra-processed foods) and adding some extra items (e.g. Plant milk and soy yogurt). The technicians requested to the adolescents to report the average frequency of consumption for the specified standard units or portion size for each food item of the FFQ. The questionnaire included 9 frequency categories for each item ranging from “never or < 1 time/month’’ to ‘‘≥6 times/day” (Supplementary Table 1). All the selected frequency categories for each food item were converted to a daily intake.

Several primary endpoints concerning the neuropsychological function of adolescents were assessed at baseline (pre-intervention). The administration of all neuropsychological tests was carried out at the school by one trained psychologist and two fieldwork technicians. Similar procedure was applied for anthropometric measurements. Standard techniques were used to measure height, weight, and waist circumference (SECA 214 stadiometer for height, SECA 770 weighing scales for weight, and SECA 201 tape for waist measurement).

### Primary outcomes

Based on WSS outcome data, three primary outcomes were selected for the current study. The first primary outcome was the self-reported Strengths and Difficulties Questionnaire (SDQ), a child mental health assessment tool with five hypothesized subscales. The scale was originally developed for the measurement of five aspects related to mental health screening namely four “difficulty” domains: hyperactivity/inattention, emotional symptoms, conduct problems, and peer problems. The SDQ additionally gathers data on prosocial behavior as a strength domain [28]. The SDQ externalizing score, which ranges from 0 to 20, is calculated by adding the conduct problems and hyperactivity scales. Emotional symptoms and peer problems scores are added to create the SDQ internalizing score, which has also a range of 0 to 20. In this case, we looked at the two SDQ profiles, where a higher score indicates abnormal behaviors.

The second measurement used was the Attention Network Test (ANT), a computer-based neuropsychological test to determine the attention function performance and the integrity of the three attentional networks [29, 30]: Alerting, the capacity to achieve and sustain maximum vigilance and performance while performing tasks; executive attention, which entails recognizing and resolving shifting attention to sensory inputs; and orienting, which includes shifting attention to sensory stimuli. Participants had to quickly determine the direction of the middle arrow when five arrows appeared on the screen. The impulsivity index was selected for the current research, which is calculated by deducing the reaction times in incorrect responses from the reaction times in correct responses, measured in milliseconds (ms). Lower scores indicate better attention performance (less impulsivity). The ANT impulsivity index is useful for identifying mental health changes, behavioral regulation, and risk-taking behavior in the adolescent population.

The third measurement analyzed, to assess emotional face recognition, was the Emotion Recognition Task (ERT). The neuropsychological test is a computer-generated paradigm for assessing the recognition of six fundamental facial emotional expressions: anger, contempt, fear, pleasure, sadness, and surprise. One at a time, the screen shows computer-generated images that have been warped from actual people’s facial characteristics to represent various emotions [31]. Each face is shown for 200 ms and then immediately covered up to prevent residual processing of the image. Finally, the participant must choose one of six possible emotions based on the expression on the face. A total of 60 images were used for emotion recognition. The outcome measures for ERT cover the correct total responses of facial emotions; higher scores indicate better emotional recognition.

### Reduction of dimensionality and dietary patterns

The food frequency questionnaire (FFQ) was used to identify dietary patterns performing PCA. All food items listed in the FFQ, except for water, were used to derive the PCA in daily servings. The original food items included from the Spanish questionnaire are detailed in Supplementary Table 1. PCA is a reduction of dimensionality technique useful for analyzing complex and vast data. The main idea is to reduce the number of variables and detect “intrinsic patterns” in the data based on linear combinations of questionnaire food, illustrating the combinations of food that are usually eaten together in an individual’s diet of group study [32]. Since this FFQ version has only 60 items, we preferred to proceed with this agnostic approach (PCA) without any prior item manipulation, such as regrouping the items to common nutrient domains.

The adequacy of the data for factor analysis was evaluated in advance of PCA (Supplementary Table 2). The Kaiser-Meyer-Olkin test and Bartlett’s test of sphericity were conducted before PCA analysis to observe the relationship between variables. PC factors were retained considering factors with eigenvalues greater than 1.8 (Supplementary Table 3). Then, the varimax rotation was performed on PCA to simplify the factor structure and to enhance their interpretability. The rotated component fed the factor loadings for each food question contained in the factor. To interpret the results, variables with loadings greater than |0.2| were considered to contribute significantly to the pattern. Finally, factor standardized scores were also saved, as continuous and tertiles, for each PC for posterior regression analysis.

PCA standardized scores are a standardized system for representing an individual’s position within a component. As the score increases, it indicates that the individual is more represented in the foods with higher positive loadings of the PC. Conversely, as the score decreases, it means that the individual is less represented by the positive loadings of the PC. In the case of a component with negative loadings (i.e., PC1 and PC5), as the score increases (positive values), it indicates that the individual is more represented by eating less food with negative values.

### Statistical analysis

After the creation of the PCA derived dietary patterns, multivariate linear regression models were performed to study the association between PCA standardized scores (continuous and tertiles) and neuropsychological outcomes (SDQ externalizing score, SDQ internalizing, ANT impulsivity index, ERT score), and all final models were adjusted for confounding variables.

First, we performed the multivariate linear regressions between PCA standardized scores and neuropsychological outcomes to study their linear association. Then, the PCA standardized scores were divided into three tertiles to avoid potential problems with non-linear relationships between the exposure and the outcome. By converting the tertiles into continuous variables (coded as 1, 2, and 3), we calculated the p-for-trend to evaluate the dose-response effect of the exposure variable. The confounding variables included in the regression models were as follows: age, gender, body mass index (BMI), physical activity and maternal education. Age was studied as a continuous variable (12–16 years old), while gender was categorical (female / male). The BMI, based on the World Health Organization (WHO) referent, was computed weight (kg)/length (m) [2] and then converted as z-scores (BMI z-scores). Physical activity was evaluated as high-intensity activity practice in four categories: once, twice, three times and more than three times a week. Finally, maternal education (presence or absence of university studies) was expressed as dichotomous.

For all regression models, the p-value of < 0.05 was considered statistically significant. Specifically, in the analysis of the ordinal exposure variable (categories), we considered the test as p for trend, with p-values < 0.05 indicating statistical significance. We did not conduct an in-depth analysis when significance was found only in the categorical exposure variable without the presence of a significant p for trend. To reduce the Type I error rates when performing multiple regression models, we conducted the False Discovery Rate test. We considered a threshold value of 0.05 to calculate the q-values. Only p-values below the q-values were considered as new thresholds for statistical significance. All analyses were conducted using RStudio version 4.2.3.

## Results

The baseline characteristics of the study population are shown in Table 1. Participants had an equal gender distribution, and age was within few years of rank (mean, 13.8 years, SD = 0.9). Most mothers’ participants had university studies (60.5%).

**Table 1:** Baseline characteristics of the study population (*n* = 643)

Variable	Value
*Adolescents*	
Female gender, N (%)	350 (54.43)
Age (years)	13.83 ± 0.94
Weight (kg)	54.32 ± 11.30
Height (m)	1.62 ± 0.09
BMI (kg/m^2^)	20.58 ± 3.45
BMI adjusted by age (z-score)	0.33 ± 1.06
Physical activity (> 3 times/week)	191 (30.07)
SDQ – Externalizing^a^	6.31 ± 3.29
SDQ – Internalizing^a^	4.22 ± 2.69
ANT - Impulsivity Index^a, b^	61.19 ± 151.4
Benton Emotional Recognition Task	48.64 ± 5.96
*Mother*	
University Education, N (%)	389 (60.59)

*BMI* Body Mass Index, *SDQ* Strengths and Difficulties Questionnaire, *ANT* Attention Network Test

Data are expressed as mean ± standard deviation, unless otherwise stated

^a^Higher score indicates worse neuropsychological performance

^b^Data in *n* = 476 participants

Six dietary patterns were extracted using PCA; eigenvalue > 1.8 criteria for PC retention and loadings greater than |0.2| were statistically relevant for the analysis. The six PC retained account for 30.77% of the total variance of the FFQ data.

**Fig. 1:**
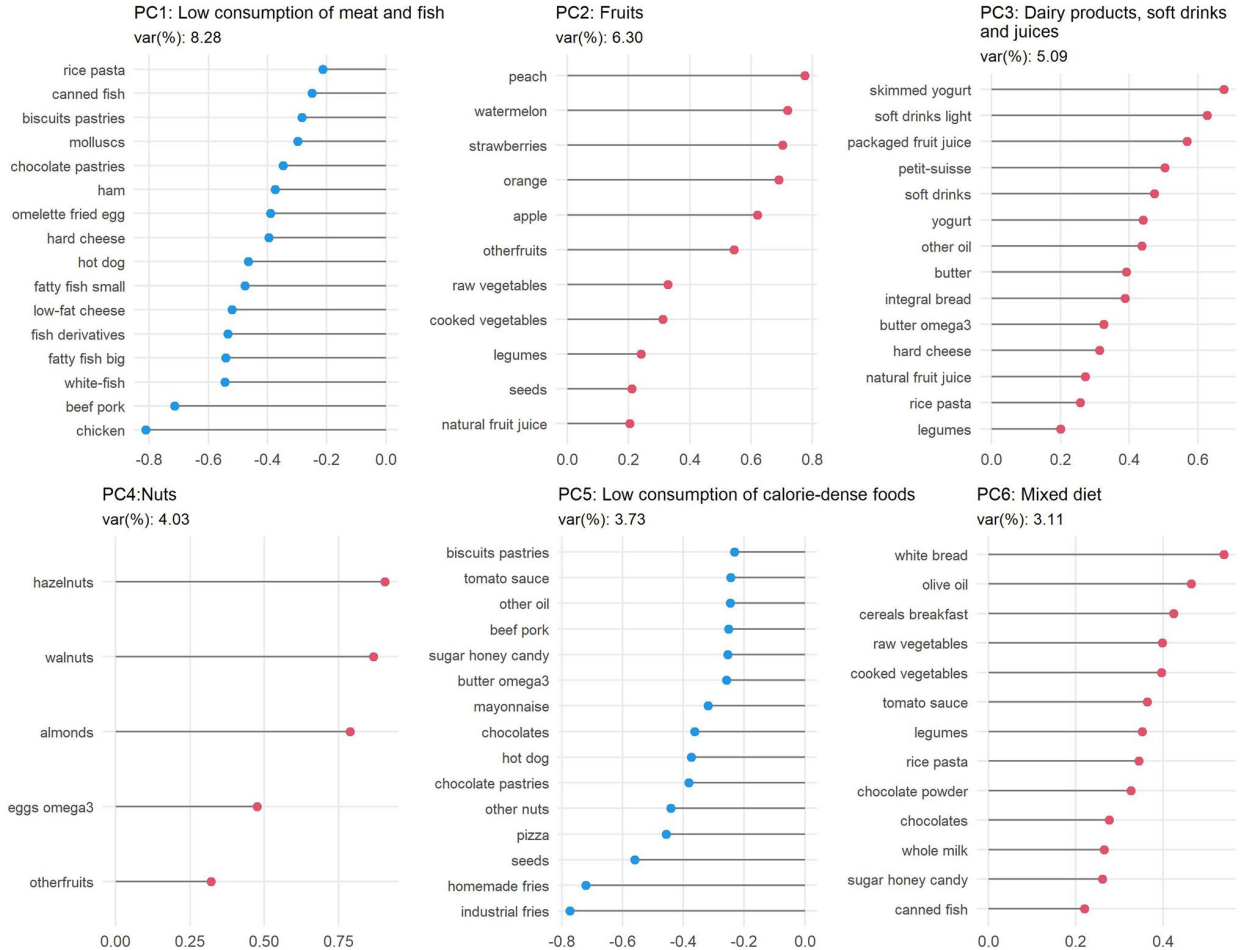
Factor loadings for the 6 principal components in adolescents PC Principal Component, var (%) proportion of variance. The 6 principal components retained, before to varimax rotation, add up to 30.77% of the total variance of the dietary data (cumulative variance)

Factor loadings for the dietary patterns are presented in Fig. 1. The first dietary pattern, referred to as the “low consumption of meat and fish” dietary pattern, was characterized by lower intake of chicken, beef pork, fish, and fish derivates, low-fat cheese, hot dog, hard cheese, omelet, fried egg, and ham. The second dietary pattern, described as “fruits” dietary pattern, was characterized by higher intake of peach, watermelon, strawberries, orange, apple, vegetables, and legumes. The third dietary pattern, referred to as the “dairy products, soft drinks and juices” pattern, was characterized by higher intake consumption of skimmed yoghurt, light soft drinks, packaged fruit juice, petit Suisse, soft drinks, other oil, butter, and integral bread. The fourth dietary pattern, referred to as the “nuts” dietary pattern, was characterized by a higher intake of hazelnuts, walnuts, almonds, omega 3 eggs, and fruits. The fifth dietary pattern, implied to as “low consumption of calorie-dense foods” pattern, was characterized by lower intake of industrial (french fries from fast-food restaurants, frozen or bagged) and homemade fries (homemade fried or cooked potatoes), seeds and other nuts, pizza, chocolate pastries, hotdogs, chocolates, and mayonnaise. The sixth dietary pattern, described as a “mixed” dietary pattern, was characterized by a higher intake of white bread, olive oil, cereals breakfast, raw vegetables, cooked vegetables, tomato sauce, legumes, and rice pasta.

Table 2 presents the fully adjusted model associations between patterns of diet and neuropsychological outcomes in the adolescents’ population. For SDQ externalizing score, statistically significant inverse associations were found for “low consumption of calorie-dense foods” as continuous ($\:{\beta\:}_{1}$= −0.49, 95% CI = −0.75, −0.24, p value < 0.001) and tertiles (tertile 3; $\:{\beta\:}_{1}$= −0.93, 95% CI = −1.56, −0.30; p for trend = 0.004). The SDQ internalizing score only showed an inverse association between the “low consumption of calorie-dense foods” dietary pattern when this variable was expressed as continuous ($\:{\beta\:}_{1}$= −0.46, 95% CI= −0.67, −0.26, p value < 0.001).

**Table 2:** Multiple linear regression models for principal component (PC) dietary patterns and SDQ externalizing and SDQ internalizing outcomes in the adolescent population

	SDQ Externalizing				SDQ Internalizing			
PC	n	$\:\beta\:$^a^	95% CI^a^	*P* value	n	$\:\beta\:$^a^	95% CI^a^	*P* value
PC1 “Low consumption of meat or fish”								
Continuous	623	−0.01	(−0.27, 0.24)	0.899	623	−0.10	(−0.31, 0.10)	0.318
Tertile 1	210	Ref.		0.383^c^	210	Ref.		0.674^c^
Tertile 2	206	0.20	(−0.42, 0.84)		206	0.19	( −0.31, 0.70)	
Tertile 3	207	−0.28	(−0.91, 0.35)		207	0.10	(−0.40, 0.61)	
PC2 “Fruits”								
Continuous	623	0.01	(−0.25, 0.25)	0.990	623	−0.11	(−0.31, 0.09)	0.278
Tertile 1	206	Ref.		0.682^c^	206	Ref.		0.197^c^
Tertile 2	207	−0.05	(−0.69, 0.58)		207	−0.04	(−0.55, 0.46)	
Tertile 3	210	0.13	(−0.50, 0.76)		210	−0.33	(−0.84, 0.17)	
PC3 “Dairy products, soft drinks and juices”								
Continuous	623	−0.04	(−0.30, 0.21)	0.731	623	−0.06	(−0.27, 0.13)	0.516
Tertile 1	206	Ref.		0.135^c^	206	Ref.		0.504^c^
Tertile 2	210	−0.65^b^	(−1.28, −0.01)		210	−0.06	(−0.57, 0.44)	
Tertile 3	207	−0.49	(−1.13, 0.15)		207	0.17	(−0.34, 0.68)	
PC4 “Nuts”								
Continuous	623	−0.07	( −0.33, 0.18)	0.560	623	0.02	(−0.18, 0.23)	0.819
Tertile 1	209	Ref.		0.066^c^	209	Ref.		0.545^c^
Tertile 2	209	−0.65^b^	(−1.28, −0.01)		207	−0.10	(−0.61, 0.40)	
Tertile 3	207	−0.59	(−1.22, 0.04)		207	−0.15	(−0.66, 0.35)	
PC5 “Low consumption of calorie-dense foods”								
Continuous	623	−0.49	(−0.75, −0.24)	< 0.001	623	−0.46^c^	(−0.67, −0.26)	< 0.001
Tertile 1	208	Ref.		0.004^c^	208	Ref.		0.109^c^
Tertile 2	205	0.17	(−0.45, 0.80)		205	0.02	(−0.48, 0.53)	
Tertile 3	210	−0.93^b^	(−1.56, −0.30)		210	−0.41	(−0.92, 0.09)	
PC6 “Mixed diet”								
Continuous	623	0.09	(−0.16, 0.36)	0.463	623	−0.13	(−0.34, 0.08)	0.230
Tertile 1	206	Ref.		0.223^c^		Ref.		0.919^c^
Tertile 2	208	−0.16	(−0.81, 0.48)		206	−0.07	(−0.59, 0.44)	
Tertile 3	210	0.40	(−0.25, 1.05)		208	0.02	(−0.50, 0.55)	

*SDQ* Strengths and Difficulties Questionnaire, *PC* Principal Component, *n* number of subjects with available data, *CI* Confidence Interval, *Ref* Reference group, *P* for trend with PC tertiles in continuous (1,2 and 3)

^a^Beta coefficient and 95% CI estimated using multiple linear regression models adjusted for sex, age, BMI z-score, physical activity, and maternal education. The regression for the exposure has been computed in continuous values and in tertiles

^b^p-value < 0.05 compared to referent tertile

^c^The p value is P for trend. The p for trend has been obtained when the regression takes the tertile values in numerical categories (1, 2 and 3)

Table 3 shows the fully adjusted model associations between the PC dietary patterns and the neuropsychological outcomes (ANT impulsivity index and ERT score). ANT impulsivity index showed an inverse association with “nuts” dietary pattern when this exposure was continuous ($\:{\beta\:}_{1}$= −24.60, 95% CI = −36.80, −12.41, p value < 0.001). We also observed a positive association between ERT score and “low consumption of calorie-dense foods” dietary pattern as continuous standardized score ($\:{\beta\:}_{1}$= 0.59, 95% CI = 0.14, 1.04, p value = 0.010) and tertiles (tertile 3: $\:{\beta\:}_{1}$= 1.31, 95% CI = 0.18, 2.44, p value < 0.05; p for trend = 0.023).

**Table 3:** Multiple linear regression models for principal component (PC) dietary patterns and ANT Impulsivity Index and ERT outcomes in the adolescent population

	ANT Impulsivity Index				ERT			
PC	n	$\:\beta\:$^a^	95% CI^a^	*P* value	n	$\:\beta\:$^a^	95% CI^a^	*P* value
PC1 “Low consumption of meat and fish”								
Continuous	469	3.06	(−9.71, 15.85)	0.637	599	−0.17	(−0.62, 0.28)	0.460
Tertile 1	164	Ref.		0.129^c^	199	Ref.		0.106^c^
Tertile 2	155	−18.77	(−52.91, 15.35)		199	−0.53	(−1.66, 0.59)	
Tertile 3	156	−26.17	(−60.09, 7.73)		201	−0.92	(−2.04, 0.19)	
PC2 “Fruits”								
Continuous	469	2.67	(−11.54, 16.88)	0.712	599	0.23	(−0.24, 0.71)	0.335
Tertile 1	163	Ref.		0.452^c^	200	Ref.		0.251^c^
Tertile 2	149	36.18^b^	(1.89, 70.48)		197	0.62	(−0.50, 1.75)	
Tertile 3	163	12.92	(−20.41, 46.26)		202	0.65	(−0.46, 1.77)	
PC3 “Dairy products, soft drinks and juices”								
Continuous	469	0.41	(−12.59, 13.42)	0.951	599	−0.07	(−0.53, 0.37)	0.730
Tertile 1	158	Ref.		0.631^c^	201	Ref.		0.173^c^
Tertile 2	152	7.09	(−27.45, 41.65)		199	1.07	(−0.04, 2.18)	
Tertile 3	165	−8.01	(−41.94, 25.90)		199	0.78	(−0.34, 1.91)	
PC4 “Nuts”								
Continuous	469	−24.60	(−36.80, −12.41)	< 0.001	599	−0.21	(−0.65, 0.23)	0.350
Tertile 1	165	Ref.		0.373^c^	206	Ref.		0.422^c^
Tertile 2	151	−5.34	(−39.51, 28.82)		194	−0.22	(−1.34, 0.89)	
Tertile 3	159	−15.26	(−48.86 ,18.33)		199	−0.45	(−1.57, 0.66)	
PC5 “Low consumption of calorie-dense foods”								
Continuous	469	11.44	(−8.60, 31.50)	0.263	599	0.59	(0.14, 1.04)	0.010
Tertile 1	161	Ref.		0.650^c^	203	Ref.		0.023^c^
Tertile 2	154	−0.98	(−35.32, 33.35)		197	0.26	(−0.85, 1.38)	
Tertile 3	160	7.87	(−26.25, 42.01)		199	1.31^b^	(0.18, 2.44)	
PC6 “Mixed diet”								
Continuous	469	3.45	(−10.72, 17.63)	0.632	599	0.27	(−0.20, 0.75)	0.256
Tertile 1	151	Ref.		0.969^c^	200	Ref.		0.717^c^
Tertile 2	165	−19.73	(−54.28, 14.82)		197	0.41	(−0.72, 1.56)	
Tertile 3	159	0.03	(−35.14, 35.20)		202	0.21	(−0.93, 1.37)	

*ANT* Attention Network Test for Impulsivity Index, *ERT* Emotional Recognition Tasks, *PC* Principal Component, *n* number of subjects with available data, *CI* Confidence Interval, *Ref* Reference group, *P* for trend with PC tertiles in continuous (1, 2 and 3)

^a^Beta coefficient and 95% CI estimated using multiple linear regression models adjusted for sex, age, BMI z-score, physical activity, and maternal education. The regression for the exposure has been computed in continuous values and in tertiles

^b^p-value < 0.05 compared to referent tertile

^c^The p-value is P for trend. The p for trend has been obtained when the regression takes the tertile values in numerical categories (1, 2 and 3)

We performed the False Discovery Rate test for each regression model. Finally, after adjusting the new comparisons of p-values with q-values, the condition to accept the alternative hypothesis was met only in the following regressions when the exposure variable is continuous: SDQ externalizing and PC5 “low consumption of calorie-dense foods” (p value < 0.001, q value = 0.006), SDQ internalizing (p value < 0.001, q value = 0.002), and PC5 “low consumption of calorie-dense foods” and Impulsivity Index and PC4 “Nuts (p value < 0.001, q value = 0.004)” (Supplementary Table 4). The regression model where the exposure variable (PC) was in ordinal tertiles 1, 2, and 3 (p for trend) did not meet the conditions of the Benjamini-Hochberg critical value (Supplementary Table 5).

## Discussion

In this cross-sectional study conducted within the framework of the WSS Intervention Trial [24], we observed the association between PCA-derived dietary patterns and neuropsychological outcomes in the adolescent population. Six dietary patterns were identified that demonstrate specific characteristics: “low consumption of meat and fish”, “fruits”, “dairy products, soft drinks and juices”, “nuts”, “low consumption of calorie-dense foods” and “mixed diet”. In fully adjusted multivariable models, “low consumption of calorie-dense foods” dietary pattern was negatively associated with SDQ externalizing and internalizing problems. Additionally, the “nuts” dietary pattern was negatively associated with the ANT impulsivity index. All these beneficial associations between diet and neuropsychological outcomes remained significant after FDR corrections.

According to neuropsychological outcomes, we found that “low consumption of calorie-dense foods” dietary patterns had a protective association with SDQ externalizing problem score, which includes ADHD symptoms and conduct problems. Other observational studies have shown an association between ultra-processed and junk food with externalizing symptoms and mental health disorders in children and adolescents [33, 34]. Independent of other potential confounding variables and childhood diet, higher intakes of unhealthy foods in children, even in pregnancy [35], predicted externalizing problems. The Western Australian Pregnancy Cohort (Raine) Study provides more evidence for adolescent women consuming a Western dietary pattern and externalizing problem behaviors [36]. On the other hand, a cross-sectional study showed that a “low fat eating” pattern made adolescents less likely to have hyperactivity symptoms and mental disorders [37]. In like manner, we found statistically significant and inverse associations between the consumption of “low consumption of calorie-dense foods” dietary preference and SQD internalizing scores. That means that people who had low consumption of calorie-dense foods had fewer internalizing symptoms (emotional symptoms and peer problems). A meta-analysis with a child and adolescent population (age = 3.9–18 years) that considered twenty-six cross-sectional studies, and, in one case-control study, the authors described an association between unhealthy diets (i.e., junk food, fast food and food associated with takeout services) and internalizing symptoms as well as symptoms of depression [38].

Further, the adherence to the “nuts” dietary pattern showed a protective association with ANT impulsivity index. Nuts are rich in omega-3 alpha-linolenic acid (ALA). In this context, a previous cross-sectional study involving the WSS cohort observed a beneficial association between erythrocyte membrane ALA levels in tertiles and a decreasing impulsivity index [39]. Impulsive behavior in the context of attention tasks is characteristic of ADHD symptoms. In this context, other studies in children and adolescents have reported an inverse association between a healthy diet (nuts, vegetables, olive oil) with symptoms of ADHD [40, 41]. These findings can be explained because nuts are rich in omega-3 fatty acids. Omega-3 is essential for protecting neurons from oxidative stress, promoting the formation of neurites, improving synaptic activity, and promoting dendritic branching [42]. Additionally, it has been suggested that omega-3 fatty acids help in impulsive and aggressive behaviors by enhancing the regulation of hormones and neurotransmitters [43].

In statistical terms, we observed discrepancy between the significant results in the continuous PCA score and the non-significant results in the tertile analysis for the same associations (e.g., “low consumption of calorie-dense foods” PC and SDQ internalizing score; “nuts” PC and ANT Impulsivity Index) warrants careful interpretation. Continuous variables capture the complete range of data, which may make them more sensitive to detecting subtle relationships. In contrast, tertile categorization simplifies the data into broader groups, which provides wider conclusions for each representation group, respectively, to each dietary pattern.

The main limitation of this cross-sectional study is that because both the outcome and the exposure are looked at the same time, it is impossible to discern the temporal relationship between them. The inability to prove the directionality of relationships, indeed, to infer causality of the study findings, and a non-randomized design are other limitations of this cross-sectional investigation using regression models. Additionally, the sample used in this research is derived from an intervention study and it is not nationally or regionally representative. Consequently, the characteristics of our study sample may differ from those of the general population, which could impact the generalizability of our findings. Additionally, despite efforts to control for confounding factors, residual confounding remains a key issue in observational studies, and interactions with other environmental factors may not be fully accounted for. Therefore, there may be unmeasured variables that influence both dietary patterns and neuropsychological outcomes (e.g., socioeconomic status). For instance, using the mother’s education level alone may not encompass all aspects of the socioeconomic background of the WSS sample. Furthermore, we cannot discard the possibility of a certain degree of misclassification from FFQ variables and residual confounding typically described in observational studies. PCA was performed in the adolescent’s food preferences and this technique, as an advantage, condenses a bigger collection of correlated variables into a smaller number of more understandable axes of variation. However, this methodology has limitations, such as linear relationship assumptions between variables and loss of information in the dimensionality reduction process. Finally, given the use of questionnaires to collect information on the possible confounders and the non-administration of missing data in the PCA’s matrixes, the sample size was reduced in the regression models due to missing responses. However, we found statistically significant associations, even after FDR corrections, despite a relative loss of sample size.

Overall, our results suggest that dietary patterns that includes “nuts” and “low consumption of calorie-dense foods” are associated with lower symptoms of externalizing and internalizing problems, and lower impulsivity index in teenagers. These are important neuropsychological outcomes related to brain maturity during adolescence. More randomized interventional studies among teenagers are needed to determine the causality of these associations in this observational study. However, this study provides valuable insights and a scientific foundation for further investigating the effect of dietary patterns on neuropsychological functioning during adolescence.

## Supplementary Information

Below is the link to the electronic supplementary material.

Supplementary material 1 (DOCX 42.6 kb)

## Data Availability

No datasets were generated or analysed during the current study.
