# Supplementary material for: Original article: adolescent dietary patterns derived using principal component analysis and neuropsychological functions: a cross-sectional analysis of Walnuts Smart Snack cohort
Source: Eur Child Adolesc Psychiatry. 2024 Sep 18;34(5):1569–78. doi: 10.1007/s00787-024-02577-6 (PMC12122558; doi:10.1007/s00787-024-02577-6)
Supplement: Supplementary file 1 — Supplementary material 1 (DOCX 42.6 kb) [file 787_2024_2577_MOESM1_ESM.docx]

**Supplementary Information.**

**Supplementary table 1.** Food Frequency Questionnaire in the original version (in Spanish) used in the WSS cohort (number of items =60).

| **ALIMENTOS (Lácteos, carnes, pescados y derivados)** | Nunca o menos de 1 mes | 1-3 por mes | 1 por semana | 2-4 por semana | 5-6 por semana | 1 por día | 2-3 por día | 4-5 por día | 6 o más día |
| --- | --- | --- | --- | --- | --- | --- | --- | --- | --- |
| 1. Leche entera (1 vaso) |  |  |  |  |  |  |  |  |  |
| 2. Leche semi, desnatada (1 vaso) |  |  |  |  |  |  |  |  |  |
| 3. Leche enriquecida con omega-3 (1 vaso) |  |  |  |  |  |  |  |  |  |
| 4. Yogur y batidos (enteros, azucarados, sabores, líquidos) (1 vaso o unidad) |  |  |  |  |  |  |  |  |  |
| 5. Yogur desnatado (unidad) |  |  |  |  |  |  |  |  |  |
| 6. *Petit suisse*, natillas, flan, helado de crema (unidad, bola pequeña) |  |  |  |  |  |  |  |  |  |
| 7. Queso curado, semi- o cremoso (2 lonchas o trozo) |  |  |  |  |  |  |  |  |  |
| 8. Queso bajo en grasa, requesón, fresco (2 lonchas, trozo o terrina pequeña). |  |  |  |  |  |  |  |  |  |
| 9. Bebidas vegetales: de arroz, soja, avena (1 vaso) |  |  |  |  |  |  |  |  |  |
| 10. Yogur de soja (1 unidad) |  |  |  |  |  |  |  |  |  |
| 11. Huevo de gallina frito, revuelto, cocido, en tortillas o en otros platos o recetas (unidad) |  |  |  |  |  |  |  |  |  |
| 12. Huevos de gallina enriquecidos con omega-3 (unidad) |  |  |  |  |  |  |  |  |  |
| 13. Pollo o pavo (pieza mediana, incluida hamburguesas o nuggets de pollo) |  |  |  |  |  |  |  |  |  |
| 14. Ternera, cerdo o cordero: filete, hamburguesa, longaniza, en guisos y pastas (unidad o ración mediana) |  |  |  |  |  |  |  |  |  |
| 15. Salchichas, Frankfurts y similares (unidad mediana) |  |  |  |  |  |  |  |  |  |
| 16. Embutidos, fiambres: Salchichón, chorizo, fuet, salami, chóped (2 lonchas o trozo) |  |  |  |  |  |  |  |  |  |
| 17. Jamón serrano o york (2 lonchas) |  |  |  |  |  |  |  |  |  |
| 18. Pescado blanco: merluza, lenguado, dorada (un plato o ración mediana) |  |  |  |  |  |  |  |  |  |
| 19. Pescado azul grande: pez espada, atún (filete, plato, porción mediana) |  |  |  |  |  |  |  |  |  |
| **ALIMENTOS (Lácteos, carnes, pescados y derivados)** | Nunca o menos de 1 mes | 1-3 por mes | 1 por semana | 2-4 por semana | 5-6 por semana | 1 por día | 2-3 por día | 4-5 por día | 6 o más día |
| 20. Pescado azul pequeño-mediano: boquerón, sardina, caballa, salmón (plato o ración mediana) |  |  |  |  |  |  |  |  |  |
| 21. Conservas: atún, bonito, sardinas, caballa (lata pequeña) |  |  |  |  |  |  |  |  |  |
| 22. Calamares, sepia, pulpo, mariscos, berberechos, almejas, mejillones y similares (½ ración o ½ lata) |  |  |  |  |  |  |  |  |  |
| 23. Derivados de pescado: delicias de pescado, muslitos de mar, palitos de cangrejo (dos unidades) |  |  |  |  |  |  |  |  |  |
|  |  |  |  |  |  |  |  |  |  |
| **ALIMENTOS (verduras, legumbres, frutas)** | Nunca o menos de 1 mes | 1-3 por mes | 1 por semana | 2-4 por semana | 5-6 por semana | 1 por día | 2-3 por día | 4-5 por día | 6 o más día |
| 24. Verdura cruda/ ensalada: lechuga, tomate, cebolla, pimiento, zanahoria... (un plato o ración mediana) |  |  |  |  |  |  |  |  |  |
| 25. Verdura cocinada o en puré: calabaza, berenjena, calabacín, judía verde, espinacas, zanahoria, col, coliflor, brócoli (plato o ración mediana). |  |  |  |  |  |  |  |  |  |
| 26. Legumbres: garbanzos, alubias, lentejas, guisantes (plato o ración mediana o 4 cucharadas en crudo) |  |  |  |  |  |  |  |  |  |
| 27. Naranjas o mandarinas (1 unidad)  Las consume en temporada □ o durante todo el año □?  (Marque la casilla correspondiente) |  |  |  |  |  |  |  |  |  |
| 28. Manzana, plátano, pera o uva (unidad mediana)  Las consume en temporada □ o durante todo el año □? |  |  |  |  |  |  |  |  |  |
| 29. Sandía, melón (tajada)  Las consume en temporada □ o durante todo el año □? |  |  |  |  |  |  |  |  |  |
| 30. Fresas, cerezas y ciruelas (plato pequeño postre)  Las consume en temporada □ o durante todo el año □? |  |  |  |  |  |  |  |  |  |
| 31. Melocotón, nectarina, paraguayo, albaricoque (unidad mediana)  Las consume en temporada □ o durante todo el año □? |  |  |  |  |  |  |  |  |  |
| 32. Otras frutas: kiwi, papaya, mango. (unidad pequeña o plato pequeño postre)  Las consume en temporada □ o durante todo el año □? |  |  |  |  |  |  |  |  |  |
| 33. Nueces (1 puñado o 6 nueces) |  |  |  |  |  |  |  |  |  |
| 34. Avellanas (1 puñado) |  |  |  |  |  |  |  |  |  |
| 35. Almendras (1 puñado) |  |  |  |  |  |  |  |  |  |
| 36. Otros frutos secos: cacahuetes, piñones (1 puñado o bolsa pequeña) |  |  |  |  |  |  |  |  |  |
| 37. Semillas: pipas de girasol, de calabaza, sésamo (1 puñado o bolsa pequeña) |  |  |  |  |  |  |  |  |  |

| **ALIMENTOS (pan, dulces, salsas y otros platos)** | Nunca o menos de 1 mes | 1-3 por mes | 1 por semana | 2-4 por semana | 5-6 por semana | 1 por día | 2-3 por día | 4-5 por día | 6 o más día |
| --- | --- | --- | --- | --- | --- | --- | --- | --- | --- |
| 38. Pan blanco (panecillo/barrita, 2 rebanadas de molde o 3-4 palitos o roscos) |  |  |  |  |  |  |  |  |  |
| 39. Pan integral (panecillo/barrita o 2 rebanadas de molde) |  |  |  |  |  |  |  |  |  |
| 40. Cereales desayuno - chocolate, azucarados (1 puñado, 3 cucharadas soperas o 1/2 bol de cereales) |  |  |  |  |  |  |  |  |  |
| 41. Cereales desayuno – integrales, tipo *All Bran* (1 puñado, 3 cucharadas soperas o 1/2 bol de cereales) |  |  |  |  |  |  |  |  |  |
| 42. Patatas fritas o cocinadas caseras (guarnición o ración mediana) |  |  |  |  |  |  |  |  |  |
| 43. Patatas fritas tipo *McDonalds/Burger King*, congeladas o bolsita de patatas (1 ración o bolsita pequeña) |  |  |  |  |  |  |  |  |  |
| 44. Arroz, pasta y maíz cocinado o en ensaladas (1 plato mediano, 4 cucharadas soperas en crudo) |  |  |  |  |  |  |  |  |  |
| 45. Galletas y bollería -donuts, croissants, pasteles, tartas (2 galletas Maria, unidad o porción) |  |  |  |  |  |  |  |  |  |
| **ALIMENTOS (pan, dulces, salsas y otros platos)** | Nunca o menos de 1 mes | 1-3 por mes | 1 por semana | 2-4 por semana | 5-6 por semana | 1 por día | 2-3 por día | 4-5 por día | 6 o más día |
| 46. Galletas y bollería con rellenos de chocolate –*ej. bollicao* (2 galletas; unidad o porción) |  |  |  |  |  |  |  |  |  |
| 47. Chocolates, bombones y cremas de chocolate- ej. *Nocilla* (2 onzas, 2 bombones, untada bocadillo o rebanada) |  |  |  |  |  |  |  |  |  |
| 48. Chocolate en polvo, *Cola-cao* y similares (1 cucharada sopera) |  |  |  |  |  |  |  |  |  |
| 49. Azúcar, miel, mermeladas, caramelos y chucherías (1 cucharada de postre o unidad) |  |  |  |  |  |  |  |  |  |
| 50. Aceite de oliva añadido en la mesa a ensalada, pan y a platos (1 cucharada sopera) |  |  |  |  |  |  |  |  |  |
| 51. Otros aceites grasas vegetales (añadido en mesa): girasol, maíz (1 cucharada sopera) |  |  |  |  |  |  |  |  |  |
| 52. Mantequilla o margarina añadida al pan o comida (1 cucharada de postre, *minibrick* o 2 untadas) |  |  |  |  |  |  |  |  |  |
| 53. Mantequilla o margarina añadida al pan o comida enriquecida con Omega-3 (1 cucharada de postre, *minibrick* o 2 untadas) |  |  |  |  |  |  |  |  |  |
| 54. Mayonesa (o *alioli*) en ensaladas y otros platos (1cucharada) |  |  |  |  |  |  |  |  |  |
| 55. Salsa de tomate frito añadida a platos, pastas (2-3 cucharadas) |  |  |  |  |  |  |  |  |  |
| 56. Pizza, croquetas, empanadillas y similares (1 porción, 1 ración mediana o 2 unidades) |  |  |  |  |  |  |  |  |  |
| 57. Refrescos azucarados de cola, naranja o limón (1 vaso) |  |  |  |  |  |  |  |  |  |
| 58. Refrescos sin azúcar, bajos en calorías, *light*, zero, de cola, naranja o limón (1 vaso) |  |  |  |  |  |  |  |  |  |
| 59. Zumos de frutas naturales (1 vaso) |  |  |  |  |  |  |  |  |  |
| 60. Zumos de frutas envasados (1 vaso o *brick* pequeño) |  |  |  |  |  |  |  |  |  |
| *¿Consume algún otro alimento/bebida al menos una*  *vez a la semana? Añádalo a continuación.* |  |  |  |  |  |  |  |  |  |
| 61._ _ _ _ _ _ _ _ _ _ _ _ _ _ _ _ _ _ _ _ _ _ _ _ _ _ _ _ _ _ _ _ _ |  |  |  |  |  |  |  |  |  |
| 62._ _ _ _ _ _ _ _ _ _ _ _ _ _ _ _ _ _ _ _ _ _ _ _ _ _ _ _ _ _ _ _ _ |  |  |  |  |  |  |  |  |  |
| 63._ _ _ _ _ _ _ _ _ _ _ _ _ _ _ _ _ _ _ _ _ _ _ _ _ _ _ _ _ _ _ _ _ |  |  |  |  |  |  |  |  |  |
| 64._ _ _ _ _ _ _ _ _ _ _ _ _ _ _ _ _ _ _ _ _ _ _ _ _ _ _ _ _ _ _ _ _ |  |  |  |  |  |  |  |  |  |
|  | | | | | | | | | |

**Supplementary Table 2.** Bartlett’s test of sphericity and Kaiser-Meyer-Olkin factor adequacy in food frequency questionnaire.

| **Test** | | **Value** | **Interpretation** |
| --- | --- | --- | --- |
| Bartlett’s test of sphericity | Chi-square | 9481.10^*^ | The data is suitable for PCA |
| Kaiser-Meyer-Olkin factor adequacy | Overall MSA | 0.72 | 0.8 and 1 indicate the data is suitable |

PCA principal component analysis.

^*^ p-value <0.05.

**Supplementary Table 3.** Components, eigenvalues, and cumulative variance in principal components analysis (PCA).

| **Component** | **Eigenvalues** | **%Var** | **Cumulative %Var** |
| --- | --- | --- | --- |
| 1 | 4.96 | 8.28 | 8.28 |
| 2 | 3.78 | 6.30 | 14.58 |
| 3 | 3.05 | 5.09 | 19.68 |
| 4 | 2.54 | 4.23 | 23.92 |
| 5 | 2.24 | 3.73 | 27.65 |
| 6 | 1.87 | 3.11 | 30.77 |
| 7 | 1.70 | 2.84 | 33.66 |
| 8 | 1.64 | 2.27 | 36.36 |
| … |  |  |  |
| 60 | 0.13 | 0.00 | 100.00 |

**Supplementary Table 4.** Multivariate regression models when exposure is in continuous (Table 3 and 4) corrected p-values for multiple testing using the Benjamini-Hochberg false discovery rate.

| **Outcomes** | **Exposure** | **P-values** | **Rank** | **Benjamini-Hochberg critical value**  **(q values)** |
| --- | --- | --- | --- | --- |
| SDQ Internalizing^*^ | PC 5 | <0.001 | 1 | 0,002 |
| ANT Impulsivity index^*^ | PC 4 | <0.001 | 2 | 0,004 |
| SDQ Externalizing^*^ | PC 5 | <0.001 | 3 | 0,006 |
| ERT | PC 5 | 0,010 | 4 | 0,008 |
| SDQ Internalizing | PC 6 | 0,230 | 5 | 0,010 |
| ERT | PC 6 | 0,256 | 6 | 0,013 |
| ANT Impulsivity index | PC 5 | 0,263 | 7 | 0,015 |
| SDQ Internalizing | PC 2 | 0,278 | 8 | 0,017 |
| SDQ Internalizing | PC 1 | 0,318 | 9 | 0,019 |
| ERT | PC 2 | 0,335 | 10 | 0,021 |
| ERT | PC 4 | 0,350 | 11 | 0,023 |
| ERT | PC 1 | 0,460 | 12 | 0,025 |
| SDQ Externalizing | PC 6 | 0,463 | 13 | 0,027 |
| SDQ Internalizing | PC 3 | 0,516 | 14 | 0,029 |
| SDQ Externalizing | PC 4 | 0,560 | 15 | 0,031 |
| ANT Impulsivity index | PC 6 | 0,632 | 16 | 0,033 |
| ANT Impulsivity index | PC 1 | 0,637 | 17 | 0,035 |
| ANT Impulsivity index | PC 2 | 0,712 | 18 | 0,038 |
| ERT | PC 3 | 0,730 | 19 | 0,040 |
| SDQ Externalizing | PC 3 | 0,731 | 20 | 0,042 |
| SDQ Internalizing | PC 4 | 0,819 | 21 | 0,044 |
| SDQ Externalizing | PC 1 | 0,899 | 22 | 0,046 |
| ANT Impulsivity index | PC 3 | 0,951 | 23 | 0,048 |
| SDQ Externalizing | PC 2 | 0,990 | 24 | 0,050 |

SDQ Strengths and Difficulties Questionnaire, ERT Emotional Recognition Tasks, ANT Attention Network Test for Impulsivity Index.

False Discovery Rate (FDR) significance threshold was defined at 0.05. Tests are considered statistically significant if their p-values are smaller than their q-values (*).

**Supplementary Table 5.** Multivariate regression models when exposure is in continuous tertiles (Table 3 and 4) corrected p-values for multiple testing using the Benjamini-Hochberg false discovery rate.

| **Outcomes** | **Exposure** | **P-values^a^** | **rank** | **Benjamini-Hochberg critical value**  **(q values)** |
| --- | --- | --- | --- | --- |
| SDQ Externalizing | PC5 | 0,004 | 1 | 0,002 |
| ERT | PC5 | 0,023 | 2 | 0,004 |
| SDQ Externalizing | PC4 | 0,066 | 3 | 0,006 |
| ERT | PC1 | 0,106 | 4 | 0,008 |
| SDQ Internalizing | PC5 | 0,109 | 5 | 0,010 |
| ANT Impulsivity index | PC1 | 0,129 | 6 | 0,013 |
| SDQ Externalizing | PC3 | 0,135 | 7 | 0,015 |
| ERT | PC3 | 0,173 | 8 | 0,017 |
| SDQ Internalizing | PC2 | 0,197 | 9 | 0,019 |
| SDQ Externalizing | PC6 | 0,223 | 10 | 0,021 |
| ERT | PC2 | 0,251 | 11 | 0,023 |
| ANT Impulsivity index | PC4 | 0,373 | 12 | 0,025 |
| SDQ Externalizing | PC1 | 0,383 | 13 | 0,027 |
| ERT | PC4 | 0,422 | 14 | 0,029 |
| ANT Impulsivity index | PC2 | 0,452 | 15 | 0,031 |
| SDQ Internalizing | PC3 | 0,504 | 16 | 0,033 |
| SDQ Internalizing | PC4 | 0,545 | 17 | 0,035 |
| ANT Impulsivity index | PC3 | 0,631 | 18 | 0,038 |
| ANT Impulsivity index | PC5 | 0,650 | 19 | 0,040 |
| SDQ Internalizing | PC1 | 0,674 | 20 | 0,042 |
| SDQ Externalizing | PC2 | 0,682 | 21 | 0,044 |
| ERT | PC6 | 0,717 | 22 | 0,046 |
| SDQ Internalizing | PC6 | 0,919 | 23 | 0,048 |
| ANT Impulsivity index | PC6 | 0,969 | 24 | 0,050 |

SDQ Strengths and Difficulties Questionnaire, ERT Emotional Recognition Tasks, ANT Attention Network Test for Impulsivity Index.

False Discovery Rate (FDR) significance threshold was defined at 0.05. Tests are considered statistically significant if their p-values are smaller than their q-values (*).

^a^ P-values is P for trend
